# Space geodetic monitoring of engineered structures: The ongoing destabilization of the Mosul dam, Iraq

**DOI:** 10.1038/srep37408

**Published:** 2016-12-06

**Authors:** Pietro Milillo, Roland Bürgmann, Paul Lundgren, Jacqueline Salzer, Daniele Perissin, Eric Fielding, Filippo Biondi, Giovanni Milillo

**Affiliations:** 1 NASA Jet Propulsion Laboratory, California Institute of Technology, 4800 Oak Grove Drive, 91109 Pasadena (CA), USA; 2 Dept. of Earth and Planetary Science, University of California, Berkeley, 389 McCone Hall, 94720, Berkeley (CA), USA; 3 GFZ German Research Centre for Geosciences, Physics of Earthquakes and Volcanoes, Telegrafenberg, 14473 Potsdam, Germany; 4 Lyles School of Civil Engineering, Purdue University, 550 Stadium Mall Drive, 47907 West Lafayette (IN), USA; 5 University of L’Aquila, l’Aquila (AQ), Italy; 6 Italian Space Agency, Contrada Terlecchia, 75100 Matera (MT), Italy

## Abstract

We present a detailed survey of the ongoing destabilization process of the Mosul dam. The dam is located on the Tigris river and is the biggest hydraulic structure in Iraq. From a geological point of view the dam foundation is poor due to a site geology formed by alternating strata of highly soluble materials including gypsum, anhydrite, marl and limestone. Here we present the first multi-sensor cumulative deformation map for the dam generated from space-based interferometric synthetic aperture radar measurements from the Italian constellation COSMO-SkyMed and the European sensor Sentinel-1a over the period 2014–2016 that we compare to an older dataset spanning 2004–2010 acquired with the European Envisat satellite. We found that deformation was rapid during 2004–2010, slowed in 2012–2014 and increased since August 2014 when grouting operations stopped due to the temporary capture of the dam by the self proclaimed Islamic State. We model the inferred deformation using a Markov chain Monte Carlo approach to solve for change in volume for simple tensile dislocations. Results from recent and historical geodetic datasets suggests that the volume dissolution rate remains constant when the equivalent volume of total concrete injected during re-grouting operations is included in the calculations.

There is a growing concern that the Mosul dam, Iraq, is at risk of failure and consequent catastrophic flooding that could affect about 1.5 million people living near the Tigris river. Maintenance grouting to close pathways opened by water infiltrating the underlying soluble evaporite deposits has been applied since the construction of the structure in 1984^1^. After the Iraq war, a reconstruction plan by the United States government and the Iraq Ministry of Water Resources was to provide for both short- and long-term solutions to this problem^1^. A US$27 million reconstruction project to establish a more effective grouting system was attempted by the US Army Corps of engineers in 2007 with modest results toward maintaining the structural health of the dam. The Iraq Ministry of Water Resources decided to keep the dam’s water level at below 319 m above sea level, thus limiting the hydrostatic pressure on the dam foundation^1^. With the rise of the self proclaimed Islamic State (IS) the dam was captured by IS in August 2014 for a short period of time. Maintenance and cement grouting operations have ceased since then and the spillways remain blocked, raising concerns about a possible dam failure.

Here we present the first comprehensive multi-sensor cumulative deformation map for the dam generated from space-based synthetic aperture radar (SAR) measurements (Fig. 1), which reveals that parts of the dam are undergoing rapid subsidence. Deformation was rapid during 2004–2010, it slowed in 2012–2014, and has increased since August 2014 when grouting operations stopped. We used data from multiple SAR satellites^2^ to measure the deformation at the dam in great spatial and temporal detail and shed new light on the dynamics of the ongoing destabilization.

## Results

We process 62 images acquired in an ascending track geometry from the Italian Space Agency (ASI) COSMO-SkyMed (CSK) constellation of four satellites to image ground deformation during 2012–2015 and 32 images acquired in a descending geometry by the European Space Agency (ESA) Sentinel-1A sensor starting October 2014 and spanning 18 months. Six Interferograms from ESA’s Envisat satellite, from both ascending and descending geometries, were computed for monitoring the destabilization process during the period 2004–2010. A multi-temporal interferometric processing technique is applied to the CSK and Sentinel-1A phase measurements to compute two separate time-series of deformation exploiting point-like scatterers^3^,^4^. We estimate time series of the water level at the dam wall using SAR amplitude images to measure the horizontal distance between the water and the top of the dam and calibrate the results with satellite altimetry data.

**Figure 1:**
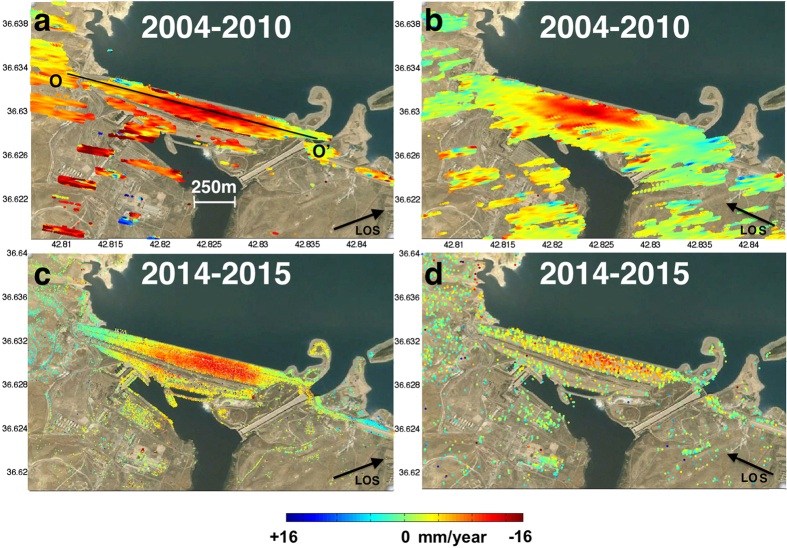
InSAR measured subsidence rates on the Mosul dam, Iraq. Negative values indicate motion away from the satellite, consistent with subsidence. (**a**) Envisat ascending track DInSAR stacked interfergrams covering May 2004–August 2010. Horizontal/Vertical Profile O-O’ is shown in Fig. 2(b) Envisat descending track DInSAR stacked interfergrams spanning May 2004–August 2010 (**c**) CSK ascending track covering December 2014–July 2015 (**d**) Sentinel descending track covering December 2014–July 2015. Time-series analysis generated using the SARPROZ Software (http://www.sarproz.com January 26 2016 Version). Map data: Google, CNES/Spot Image, Basarsoft (https://www.google.com/earth/).

**Figure 2:**
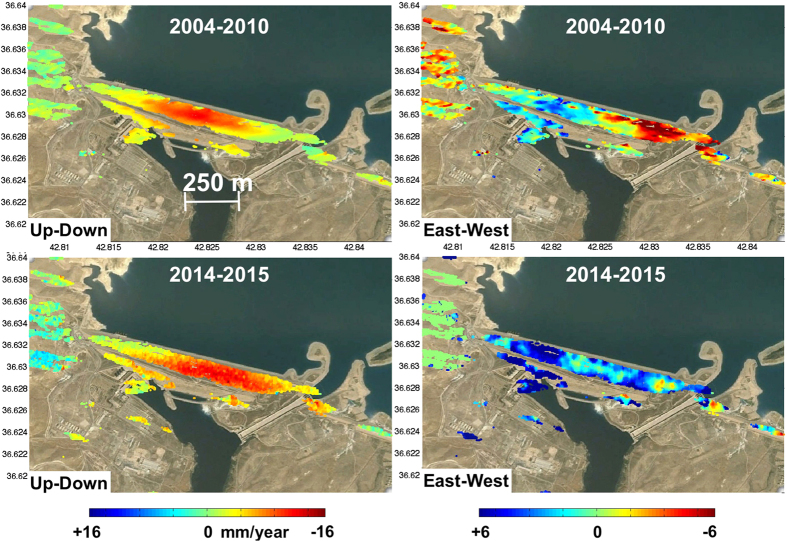
Up-down (left) and east-west(right) displacement at the Mosul dam, Iraq. Negative values indicate downward and westward motion respectively. The data have been resampled on a common grid calculated using the coherence maps from the interferograms (Envisat) and time-series analysis (CSK-Sentinel-1a). Formula (7) and (8) have been applied to the LOS displacements to calculate the up-down and east-west motion respectively. Time-series analysis generated using the SARPROZ Software ((http://www.sarproz.com. January 26 2016 Version). Map data: Google, CNES/Spot Image, Basarsoft (https://www.google.com/earth/).

**Figure 3:**
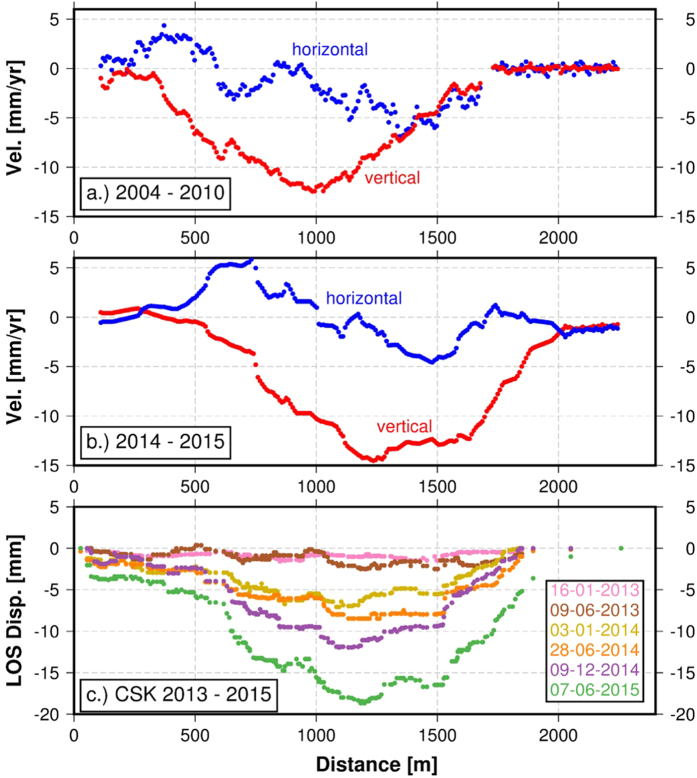
Subsidence profiles. (**a**) Vertical and east component of surface velocities along the Mosul Dam from Envisat 2004-2010 2D deformation (**b**) velocity components from CSK, Sentinel-1a velocity maps obtained using data spanning the same time period (December 2014–July 2015). The results confirm that the vertical velocities greatly exceed the horizontal contribution typical of subsidence patterns. (**c**) CSK LOS cumulative displacement profiles.

Data from the different spacecraft show that Mosul dam subsided throughout the observation period at varying rates and reveal a shift in the location of the maximum deformation magnitude. The early Envisat dataset provides a good constraint on the first-order deformation pattern (Fig. 1a,b, Figs 2 and 3a), showing subsidence with only small horizontal motions (Fig. 2). Figure 1c and d show the recent displacement rate along the LOS direction of the CSK and Sentinel-1A radar respectively over 8 months (December 2014- July 2015). The observed subsidence is likely caused by dissolution of the dam substrate by water from the reservoir penetrating into “soluble sediments consisting of eastward dipping beds and pods of anhydrite/gypsum, interbedded with layers of sand, silt and soft weathered marls, brecciated and clay-like marls, and fresh-to-weathered limestone”^5^.

**Figure 4:**
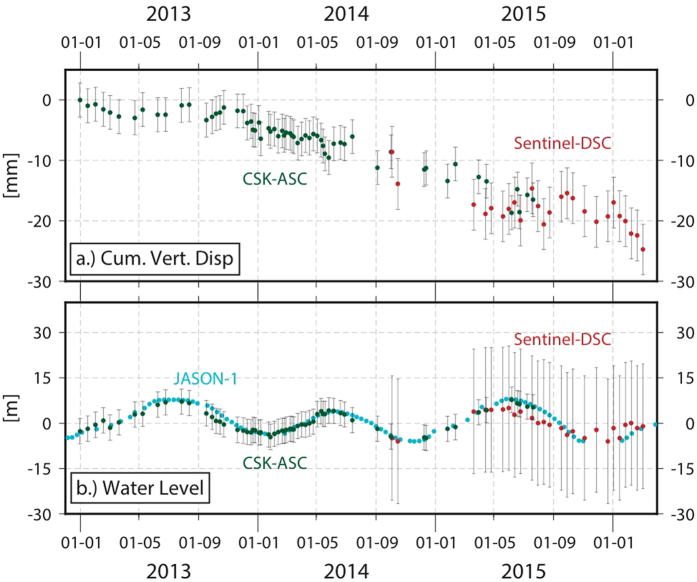
(**a**) CSK/Sentinel-1a Time series of subsidence at the center of the dam assuming negligible North-South motion (**b**) CSK/Sentinel-1a amplitude derived shoreline location on the dam face calculated from amplitude measurements calibrated with JASON-1 altimeter data.

The Envisat measurements from 2004 to 2010 indicate a maximum line-of-sight (LOS) velocity of −11.5 mm/year ± 6 mm/year (−12.47 mm/year vertical, negative rates indicate LOS increase or subsidence, Fig. 3a). The fine spatial resolution (3 m) and dense temporal sampling of the CSK constellation allow us to better constrain the transient deformation during 2012–2015. A regression analysis suggests that the dam is deforming at a maximum LOS rate of −12.1 ± 1.8 mm/year (−14.9 mm/year if considered vertical displacement) (Fig. 3b). Subsidence rates from December 2012 to December 2013 did not exceed −2.3 ± 1.8 mm/yr (Fig. 4a). Due to the polar orbit geometry of all the SAR satellites, we do not have constraints on N-S displacements that might be associated with fluctuating water levels and associated pressure changes on the dam. However, the CSK LOS time-series do not correlate with SAR-based water level measurements, suggesting only a modest contribution by elastic dam deformation due to the changing water load. The most recent Sentinel-1A data confirm the subsidence rate observed with CSK (−14.6 ± 4.3 mm/year) (Figs 1d and 4a) extending our observations until March 2016. The higher standard deviation is mainly due to the reduced number of scenes (25) increasing the noise level in the non-linear time-series estimation (see methods section for major details).

Using ascending and descending viewing geometries we can infer the horizontal (east-west component) and vertical displacement rates using images acquired during the same time interval (Fig. 2). We oversample the results on a common 3 m grid of the CSK Stripmap-himage products. The results confirm that the vertical velocities greatly exceed the horizontal contribution (Figs 2 and 3). Comparison of the 2004–2010 and 2014–2015 subsidence-rate profiles indicates a 300 m eastward shift of the peak subsidence toward the dam’s main spillway (Fig. 3). The main difference between the spatial patterns of deformation during these two periods is related to the widening of the CSK/Sentinel-1a vertical profile (Fig. 3b). The Envisat subsidence rate profile (Fig. 3a) is 5 mm/year slower compared to the CSK/Sentinel-1A profile.

## Discussion

The Mosul dam is subsiding at a linear rate of ~15 mm/year compared to 12.5 mm/year subsidence rate in 2004–2010. The subsidence restarted at the end of 2013 after re-grouting operations stopped. The causes of the observed linear subsidence process can be found in the human activities that have promoted the evaporite–subsidence development, primarily in gypsum deposits and may enable, in case of continuous regrouting stop, unsaturated water to flow through or against evaporites deposits, allowing the development of small to large dissolution cavities.

Large vertical movements have resulted from the dissolution of extensive gypsum strata previously mapped beneath the Mosul dam^5^. Increased subsidence rate has been due to the absence of regrouting underlying the dam basement. The subsidence seems to have a linear behavior but we can not exclude a future acceleration due to the increased dissolution speed.

Although a detailed continuum mechanical modeling of the observed subsidence is not the primary goal of this paper, the simplified model (supplementary information) does show the ability of the observed deformation to provide first-order constraints on the primary processes involved in the dam’s basement.

In Particular, simple models of the pattern of deformation put a rough constraint on the source depth, assuming a horizontal tensile dislocation in an elastic half-space^6^) (See auxiliary material). Even if the adopted model does not take into consideration the plasticity conditions of the rock succession, it is consistent with *in situ* measurements of water temperature made by the US military using independent measurements^5^. The slowdown of the deformation can be interpreted as being due to the reduced water level during the December 2012 to June 2013 dry season (Fig. 3b) and consequent low water pressure at the dam basement, which we suggest results in reduced dissolution rates of the evaporites underlying the dam foundation.

The inferred lateral propagation of the deformation source seems to be compatible with a hydrogeologic model^5^ constrained by ground measurements in which the dam substrate is characterized by an increased hydraulic gradient toward the east abutment: promoting fluid pathways connecting the reservoir to the downstream rocks in the subsurface. The model identifies the reservoir-induced pressure as the main cause of an increase in the dissolution rates. Moreover the abundant presence of readily soluble gypsum below the eastern dam foundation close to the main spillway seems compatible with the observed subsidence shift and the eastward deepening of the dissolution front. These observations agree with the previously mapped geometry of the sedimentary strata^5^, characterized by eastward dipping gypsum beds. SAR data analysis together with the inferred lateral propagation from knowledge of the geology and hydraulic connectivity suggest that the locus of foundation dissolution has shifted eastward toward the dam’s principal spillway.

Model Inversions for the locus of volume loss in 2004–2010 infer a depth of 70 meters below the surface and a deepening in 2014–2015 of 160 meters below the surface (see auxiliary material). The annual increase of water levels in the future, especially during the summer months, could lead to an increase of water pressure in the dam foundation, which combined with the absence of regrouting could speed up the dissolution of the dam substrate and promote its destabilization.

The Markov chain Monte Carlo (MCMC) approach^5^ enables us to calculate the dissolved volume loss rates at different times (34.1 · 10^2^ ± 9 m^3^/year during 2004–2010 vs. 44.6 · 10^2^ ± 9 m^3^/year in 2014–2015), indicating a speedup of the dissolution that was likely caused by the lack of maintenance and regrouting. To support this hypothesis, we calculate the sum of the injected cement volume rate^1^ and dissolved volume-loss rate in 2004–2010 (15 · 10^2^ m^3^/year) and compare it to the total dissolved volume in 2014–2015, when regrouting was stopped. We find that the estimates of total dissolution rates for the two observation periods agree within 10% (See auxiliary material). This volume change analysis supports the hypothesis that a roughly steady dissolution rate holds on a yearly basis. However, the joint time-series analysis of water levels and deformation on a weekly timescale points to the reduced water-level at the dam wall as a possible cause for the subsidence slow down during the period spanning December 2012–August 2014. We cannot confirm this short-term dynamic effect during the period 2004–2010 because of the lack of sufficiently frequent Envisat acquisitions.

We can summarize our conclusions in the following statements:

- This study highlights how the availability of new constellations of SAR sensors supported by historical SAR databases are enabling dam deformation monitoring in which InSAR measurements are accurate enough to measure sub-kilometer scale geologic and anthropogenic processes, providing critical information fundamental for mitigating anthropogenic hazards.
- Our observational strategy together with simple elastic models is in agreement with independent studies confirming both the depth of the past dissolution source and the eastward shift of the dissolution front that occurred after the regrouting stopped.

## Methods

We used an MT-InSAR approach on the COSMO-SkyMed stripmap and Senintel TOPS data based on a persistent scatterers (PS) interferometry processing chain. This stochastic approach^3^ has been widely adopted for monitoring deformation at dams^7^,^8^,^9^ when a copious number of acquisitions (>20) is available. The ENVISAT dataset is characterized by only 6 interfrograms, hence the PS technique is not applicable and a standard DInSAR stacking technique has been adopted (i.e. Unwrapped interferograms have been averaged and weighted with their temporal baseline).

The PS technique assumes a set of N + 1 coregistered SLC SAR images. We generate N single look differential interferograms with respect to a single master image. The master image is chosen maximizing the expected coherence of the generated interferograms following:


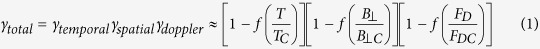



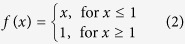


Where 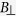, T, *F*_*D*_ and 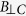, *T*_*C*_, *F*_*DC*_ are the perpendicular, temporal and doppler baselines and their critical values respectively. A first rough identification of PS is performed considering all the pixel with an inverse amplitude stability of 0.8 and choosing a pixel R relatively far from the dam as a reference point, the interferometric phase difference between two neighbors pixels *x*_*r*_ and *x*_*s*_ in the interferogram i can be written as the sum of 5 terms:


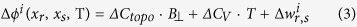


Where *ΔC*_*topo*_ and *ΔC*_*V*_ are proportional to the DEM errors and mean velocity difference between pixels *x*_*r*_ and *x*_*s*_ respectively. *Δw*_*r,s*_ contains the sum of three contributions related to atmospheric phase screen (APS) noise and non linear motion. We assume 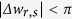 for neighbors pixels. We estimate *ΔC*_*topo*_, *ΔC*_*V*_ maximizing the absolute value of the temporal coherence 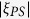 for neighbors pixels.

Where:


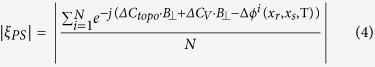


We then unwrap every interferogram estimating the the phase term 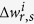.

We consider the temporal low pass and high pass component of:


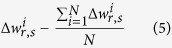


the low pass component is considered the non linear component of motion while for each interferogram a low pass spatial filter is applied on the high pass component to estimate the APS.

These steps have been performed considering only neighbor’s pixels. The same processing is then applied considering only differences between the reference point R and all persistent scatterers including the estimated APS in eq. (4).

The accuracy of the estimated velocities and DEM errors can be derived by a regression analysis taking into account the irregularly sampled data (perpendicular baseline and temporal baseline in the case of the CSK constellation. As in ref. 4:


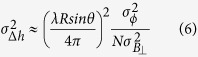



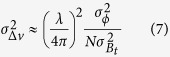


where R is the target-sensor distance, θ is the incidence angle, λ is the sensor wavelength, 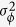 is the phase noise variance supposed independent of the acquisition and 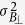, 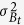 are the variance of the perpendicular and temporal baselines respectively.

MT-InSAR provides ground displacements along the LOS. Given the geometry diversification our dataset it is possible to combine ascending and descending cumulative displacement maps covering the same temporal period to highlight vertical and east-west deformations where:


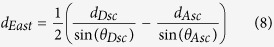



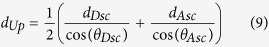


Where 39.2, 35.5 and 22.7 degrees are the incidence angles for Sentinel, CSK and ENVISAT respectively. In this formulation we considered the C-band and X-band sensors penetration depth negligible when combining ascending and descending data.

For what concern the water level estimation we manually measure in a GIS environment the distance between the top of the dam and the water level in the SAR images. Our measurements measure the pixels’ distance and estimate a scaling (slope) factor by calibrating the amplitude based water levels with the JASON-1 altimetrer. The standard errors are calculated taking into account the pixel size of the sensors (Range + Azimuth).

## Additional Information

**How to cite this article**: Milillo, P. *et al*. Space geodetic monitoring of engineered structures: The ongoing destabilization of the Mosul dam, Iraq. *Sci. Rep.* **6**, 37408; doi: 10.1038/srep37408 (2016).

**Publisher's note:** Springer Nature remains neutral with regard to jurisdictional claims in published maps and institutional affiliations.

## Supplementary Material

Supplementary Information
