# Supplementary material for: Space geodetic monitoring of engineered structures: The ongoing destabilization of the Mosul dam, Iraq
Source: Sci Rep. 2016 Dec 6;6:37408. doi: 10.1038/srep37408 (PMC5138637; doi:10.1038/srep37408)
Supplement: Supplementary Information [file srep37408-s1.doc]

**Space geodetic monitoring of engineered structures:**
**The ongoing destabilization of the Mosul dam, Iraq**

**Pietro Milillo*^1^, Roland Bürgmann^2^, Paul Lundgren^1^, Jacqueline Salzer^3^, Daniele Perissin^4^, Eric Fielding^1^, Filippo Biondi^5^ and Giovanni Milillo^6^**

^1^NASA Jet Propulsion Laboratory, California Institute of Technology, 4800 Oak Grove Drive, 91109 Pasadena (CA), USA.

^2^Dept. of Earth and Planetary Science, University of California, Berkeley, 389 McCone Hall, 94720, Berkeley (CA), USA

^3^GFZ German Research Centre for Geosciences, Physics of Earthquakes and Volcanoes, Telegrafenberg, 14473 Potsdam, Germany.

^4^Lyles School of Civil Engineering, Purdue University, 550 Stadium Mall Drive, 47907 West Lafayette (IN), USA

^5^University of L’Aquila

^6^Italian Space Agency, Contrada Terlecchia, 75100 Matera (MT), Italy

*Corresponding author [pietro0milillo@gmail.com].

**Supplementary Material**

We use a Markov chain Monte Carlo (MCMC) approach (7) to solve for change in volume of simple tensile dislocations. We divide the dislocation into 3 patches corresponding to the west, center and east part of the dam. We estimate the depth and slip (closing) of the tensile dislocations. For each parameter we estimate the probability density functions (PDF) from histograms of retained solution. We run the MCMC algorithm iteratively until reaching 1 million kept solutions. The most probable parameters are estimated from the PDF together with 95% confidence intervals. Figure 1S shows the inverted parameters for the CSK and Sentinel-1a data.

We found a stable solution after fixing the total dislocation length, width (which in our case is the dimension across-dam across-dam) and center position. When too many parameters are inverted at the same time the PDFs for several of the parameters do not form smooth, Gaussian-like distributions, but instead are multi-modal. In addition, we must note that we are using a very simple analytical solution for an elastic half-space to model a source process that is likely more complex involving a combination of both elastic and anelastic deformation processes.

The model indicates a net volume-loss rate of 3415 ± 9 m^3^/year during 2004-2010 (Envisat, Figure 2S), and 4459 ± 9 m^3^/year in 2015 (CSK, Sentinel-1a) . The formal error does not take into account systematic errors in the data or errors in the assumptions made in the model. Our model found a 90 meters difference in depth between different periods 2004-2010 (Envisat), 2015 (CSK, Sentinel-1a) as indicated by the broader subsidence pattern (Figure 2).

The total average injected volume per year of concrete during 2004-2007 (1, page 58) amounts to 1496.5 m^3^/year where a unit volume mass of 1506 kg/m^3^ has been used to convert cement mass into volume. The sum of the injected cement volume and the estimated volume loss in 2004-2010 is within 10% of the total dissolved volume in 2014-2015 when regrouting stopped.


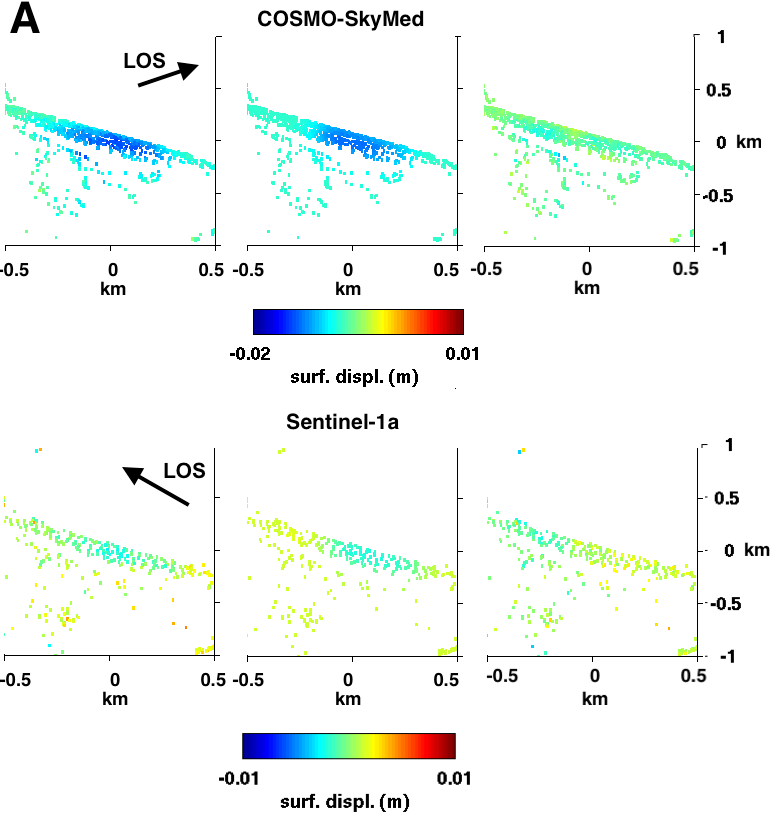


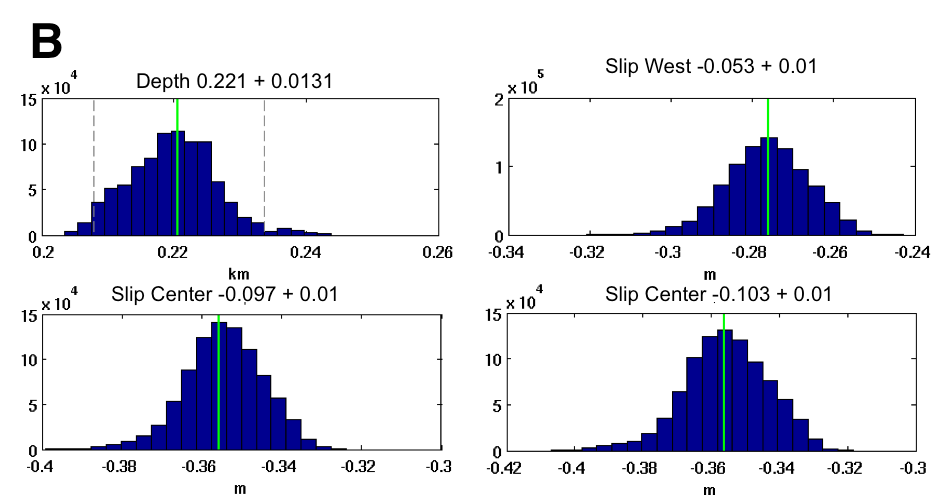


**Figure 1S. A.** Modeling results for the tensile dislocation. For each data (left), modeled synthetic (center) and the residual (right) are shown. Arrows show the satellite heading (gray) and radar look direction (black) along with the ground incidence angle. Velocity maps obtained using CSK and Sentinel-1a data spanning the same time period (2014 –2015) B. Tensile dislocation probability density distributions determined by the Markov chain Monte Carlo Bayesian inference approach. The dislocations are forced to be on the same plane (i.e. equal depth).


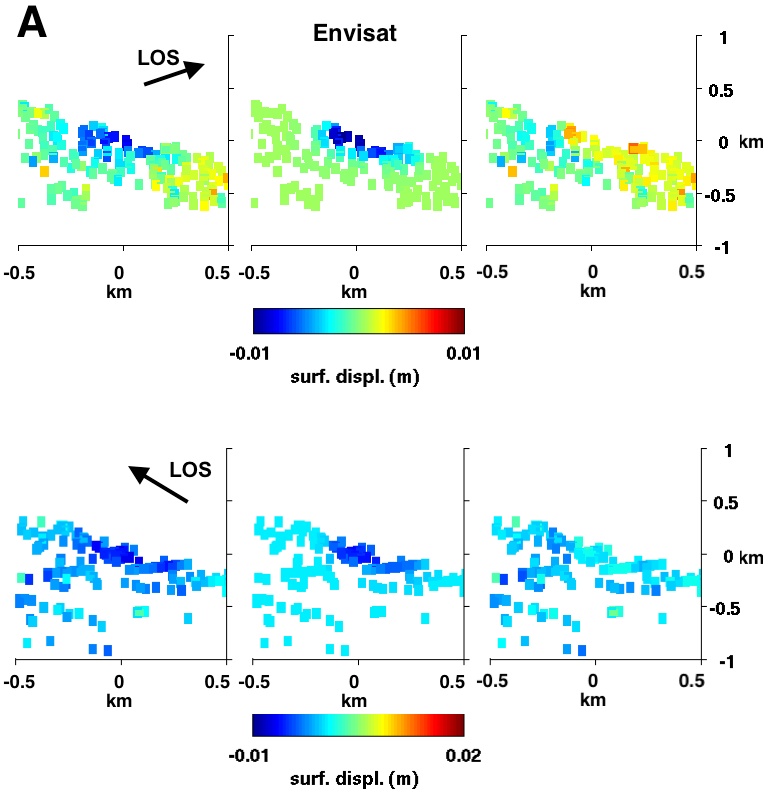


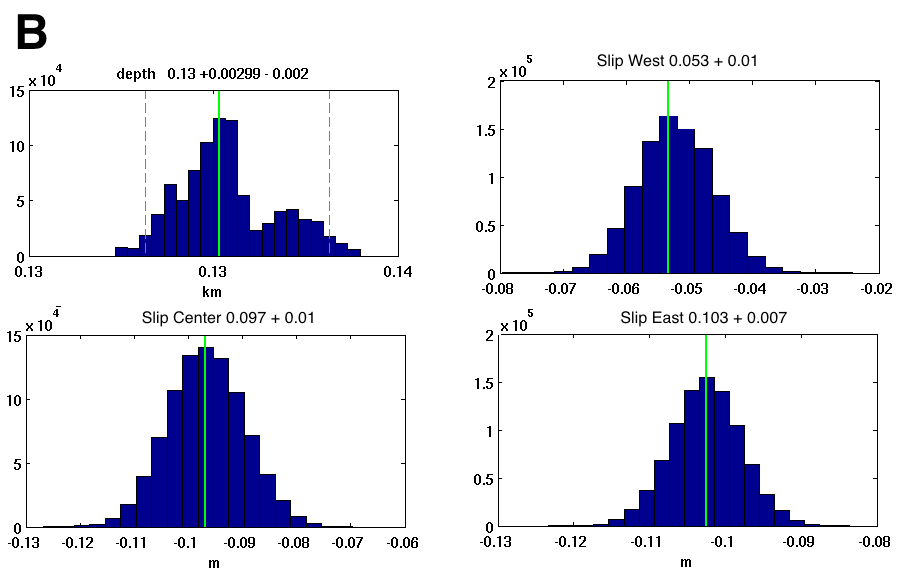


**Figure 2S**. A. Modeling results for the tensile dislocation. For each data (left), modeled synthetic (center) and the residual (right) are shown. Arrows show the satellite heading (gray) and radar look direction (black) along with the ground incidence angle. Velocity maps obtained using InSAR stack spanning the same time period (2004 –2010) B. Tensile dislocation probability density distributions determined by the Markov chain Monte Carlo Bayesian inference approach.
